# Learning From Artemisia’s Lucretia: Embodied Suffering and Interoception in Suicide

**DOI:** 10.3389/fpsyt.2020.00758

**Published:** 2020-07-31

**Authors:** Philippe Courtet, Sébastien Guillaume

**Affiliations:** ^1^PSNREC, University of Montpellier, INSERM, CHU de Montpellier, Montpellier, France; ^2^Department of Emergency Psychiatry and Acute Care, Lapeyronie Hospital, CHU Montpellier, Montpellier, France; ^3^FondaMental Foundation, Créteil, France

**Keywords:** suicide, interoception, self-awareness, art, body

## Abstract

In the painting “Lucretia,” Artemisia Gentileschi, one of the major painters of the 17^th^ century, depicts Lucretia’s suicide. This artwork empathic vision offers the spectator the apprehension of a unique phenomenon where psychological pain is transformed into self-aggression. To understand why the body becomes an object to attack, it is important to study the role of interoception and self-awareness in the suicidal process. This essay discusses how bodily representations are crucial for interacting efficiently and safely with the outside world and for establishing the sense of self. It presents some of the available evidence showing that alterations in the body representation and in the sensations perceived by it contribute to suicide. Indeed, neuroimaging studies show that social environmental factors and their biological consequences in the body (e.g., increased neuroinflammation) can alter the neural networks of suicidal behavior by increasing the sensitivity to psychological pain and the disconnection from self-awareness. Therefore, body image, sensations and awareness as well as psychological pain should be examined to improve the understanding of the dynamic interactions between body, brain, and mind that underly suicidal behavior. This conceptualization brings clinical and therapeutic perspectives in a domain where they are urgently needed.

## Introduction

**Figure 1:**
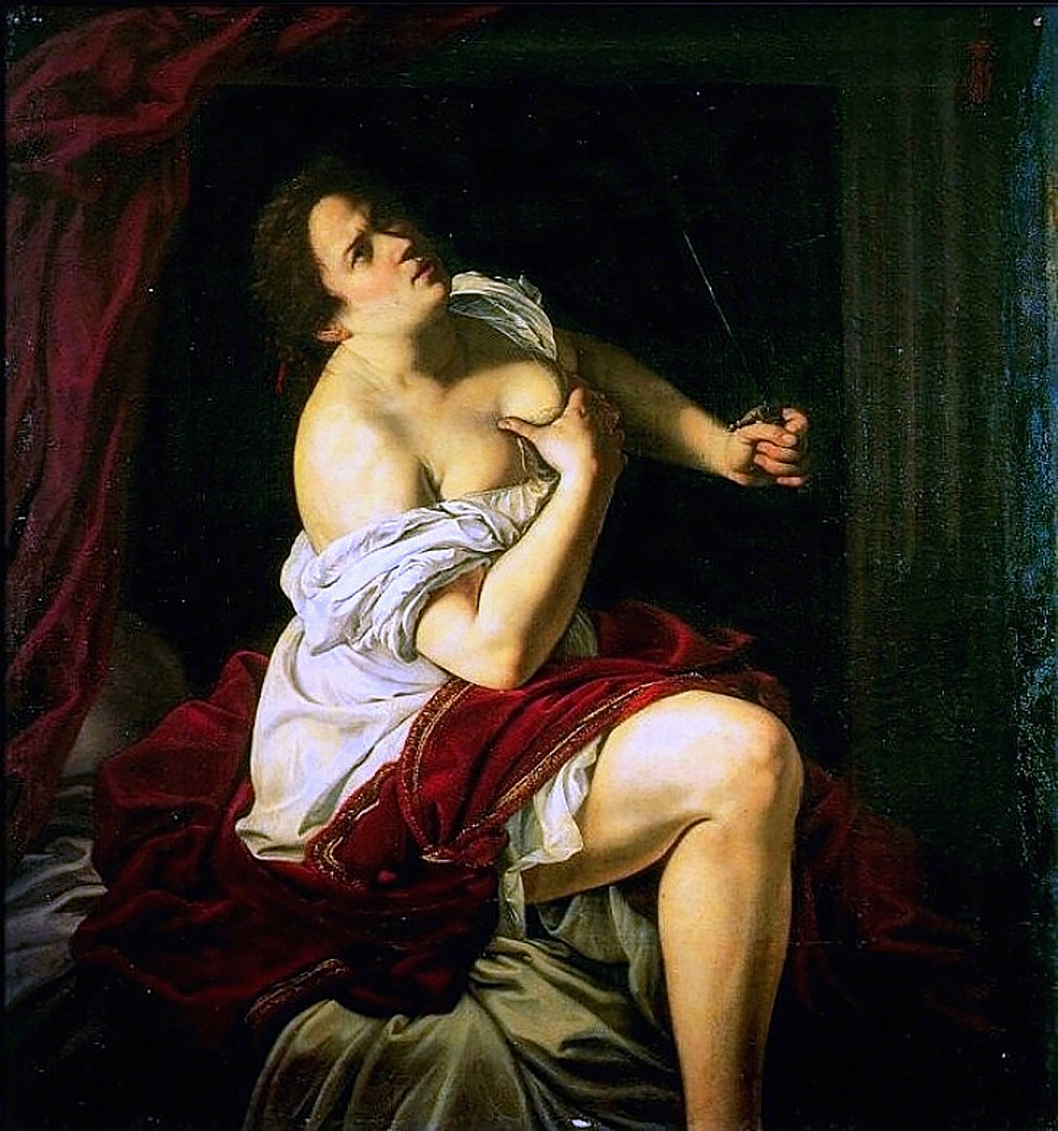
Lucretia, Artemisia Gentileschi (1). Source: Wikipedia.org

Artemisia Gentileschi (1593–1654 or later) is the most celebrated woman artist of the Baroque period in Italy. Her exceptional artwork “Lucretia’s Suicide” questions the overlooked issue of the body in suicidal behavior (**Figure 1**). More precisely, this artwork gives some insights into a rather unique process in suicidal individuals where psychic pain translates into an aggression against their own body. In this essay, our objective is, by starting from this painting, to discuss, on a narrative basis and based on recent and relevant publications, the current advances and future research directions to understand the role of interoception and self-awareness in the suicidal process.

## “Lucretia’s Suicide” by Artemisia Gentileschi

“Lucretia’s Suicide” (1) wonderfully illustrates the question of the embodied self in suicide. Lucretia was raped by the son of the Etruscan king of Rome. She then committed suicide to prove her innocence and to demonstrate her refusal to live with her honour tainted. Her rape and subsequent suicide led to the establishment of the Roman Republic. It is hard not to construe this act as an escape from social pain (e.g., fear of exclusion). Artemisia Gentileschi works have given rise to many discussions among art history experts, and brought a strong feminist perspective (2, 3). Indeed, it has been proposed that her work is a proof of her resilience because Artemisia also was a victim of rape. The depiction of Lucretia by Artemisia carries the weight of Caravaggio’s characteristic pictorial style, particularly when the painting is compared with the masterpiece by Rembrandt on the same subject (4). The remarkable physical realism and the palpable emotional turmoil of Lucretia emerging from the darkness gives a nuanced and naturalistic representation of the human condition. This also echoes a well-known text by Cardinal Paleotti (*De sacris et profanis imaginibus*, 1582), who tried to establish the Counter-Reformation doctrine on religious and secular art, where he advises painters to give in-depth instruction on the inner physiology of the human body in order to better represent the martyrs’ torments. Lucretia’s attire suggests that the rape was committed shortly before the scene depicted in the painting, and the close-focus composition accentuates the dramatic impact of the violent act. Lucretia is seized in a moment of deep contemplation, of decision making, between life and death. This is what the painting seems to tell the viewers: the dynamism in torsion that begins with the contrapposto, the Renaissance-style pyramidal architecture, the spiral drapery, and finishes with the crossed arm that goes as far as grasping the breast and indicates the dagger, which contrary to the other objects, is directed toward the sky, the same direction of her gaze. Everything in this painting leads the viewer to look at Lucrezia’s face with its expression of anguish that is manifested by the frowning eyebrows and her gaze of suicidal terror. This suicidal contemplation is accentuated by the striking contrast between the breast (reminding of the breastfeeding Virgin) and the dagger (death), which was an important issue in the feminist reading of Artemisia’s work in the 1970s. This painting captures the climax of the narrative in a simple, but deeply moving combination of facial expression, body posture, arm movements and the fateful dagger. Lucretia alone, her lonely decision made, resolved her inner conflict by ending her life. Then, why did she harm her body? Because it was harmed, as Livy told us: “But it is only the body that has been violated, the soul is pure; death shall bear witness to that.” Thanks to the art of Artemisia, the important role of pain, interoception and self-awareness in suicide is more obvious.

## Psychological Pain as Core Dimension in Suicide

The intense psychological suffering that leads people to consider killing themselves should not mask the fact that they have also a body. Indeed, people who have engaged in suicidal behavior also have somatic problems that may affect the way they see themselves (5–7). Moreover, suicidal vulnerability may be modulated by body signals. For instance, abnormalities in somatic markers (e.g., skin conductance) contribute to decision-making impairment, a cognitive trait of susceptibility to suicidal behavior, driven by the functioning of the orbitofrontal cortex (8–10).

In suicidal patients, activation of the orbitofrontal cortex is decreased during risky decision-making. This suggests that they might be more sensitive to negative emotional stimuli and short-term rewards, and consequently more prone to suicidal behaviors under the influence of pain (11). According to some prominent authors, suicide can be understood as a way to escape, through death, from a state of unbearable inner pain (12–14). Death will put an end to that pain because the *self* will disappear (15). Psychological pain is a core dimension in suicide and can predict future suicidal behavior, independently of depressive symptoms or suicidal ideation (16, 17). Psychological pain has been defined as “a lasting, untenable and unpleasant feeling that results from a negative evaluation of an incapacity or deficiency of the *self*” (18). Therefore, psychological pain can be interpreted as a “brokenness of the *self*” that induces a sense of injury, disconnection, and loss of control. Importantly, the neuroanatomy of suicide and pain involves some circuits associated with the *self*. Neuroimaging studies showed that experiences of social and psychological pain activate various important neurobiological substrates involved in suicidal vulnerability. Interestingly, some brain areas (notably insula, anterior cingulate cortex, and somatosensory cortex) are activated by psychological/social pain and also by physical pain (19), suggesting that psychological/social pain may be intimately related to bodily pain. Several studies in suicidal patients have detected difficulties in reading and interpreting emotional feedback and rewards, resulting in the activation of frontal areas upon presentation of angry faces or rewards (10, 20, 21). Altogether, the higher sensitivity to negative emotional stimuli and propension to risky decisions may lead to misinterpretations of the social environment, making suicidal patients more vulnerable to social exclusion (22). Indeed, brain activation is different in patients with and without history of suicide attempts in situations of social exclusion that induce the response of insula and anterior cingulate cortex (ACC), and therefore the response of the physical and psychological pain networks (23). Overall, as body signals modulate suicidal vulnerability, and as susceptibility to social exclusion involves a pain network, it could be hypothesized that suicidal patients have a specific neurobiology that makes them more vulnerable to attacks by their *self*.

## The Role of Interoception

Interoception is the ability to effectively perceive the physiological condition of the body, thus allowing detecting bodily sensations in a conscious way (24). Interoceptive deficits refer to a disconnection from the physical body that can cause difficulties in truly understanding and knowing their own body. Muehlenkamp & Brausch (25) theorized that interoception is an important component of body regard (how the body is perceived, cared for, and experienced). The disparity between expected and sensed interoceptive states may promote a wide range of maladaptive behaviors intended to change the internal milieu in order to match the anticipated state (26). Thus, people disconnected from their body may not feel protective and caring toward it, making easier to engage in behaviors that harm the body. Disconnection from the body may contribute to increase body objectification, which also facilitates self-harm of a body that is seen only as an object.

Various nerve pathways are responsible for detecting and mapping these homeostatic sensations (e.g., the degrees of visceral muscle contraction, the chemical composition of the internal environment). In interoception, the vagus nerve is the most important afferent pathway, and the insula is one of the main cortical targets of the interoceptive system signals (27). Insula has a pivotal role in anticipating and processing sensations to guide behavior in close connection with the ventral striatum. This provides a mechanism for the integration of interoceptive stimuli in the emotional response that generates an action or a decision (28). The activation of these brain regions, which are also involved in suicide, in pain and in social exclusion, leads to investigate the mechanisms involved in their dysregulation. Peripheral inflammation and inflammatory interoception are among the recently postulated mechanisms. Studies combining imaging techniques and induction of peripheral inflammation showed the activation of various interoceptive pathways that are projected toward the insula (29). In this way, the peripheral inflammation status is transmitted to the central nervous system (CNS) through the vagus nerve and other autonomic nerves. Similarly, some peripheral inflammation factors have been detected directly in the CNS through the circumventricular sensory organs. Moreover, some of these inflammatory mediators (interleukin-6, interferon alpha, monocytes) can cross the blood-brain barrier in small quantities. The direct trafficking of monocytes to the CNS allows the amplification of behaviors related to the stress response, by involving also the microglia. Thus, changes in the insula induced by these inflammatory interception mechanisms can contribute to changes in the subjective experience and to various psychiatric disorders.

## Inflammation and Suicidal Behavior

In suicidal behavior, growing evidence suggests the existence of a *ménage à trois* involving inflammation, social exclusion, and suicidal behavior. Social isolation, negative social interactions in daily life, and social defeat are common triggers of suicidal behavior and also strong inducers of inflammatory responses. For instance, Slavich et al. (30) reported that exposing humans to a laboratory social stress task leads to an increase in peripheral cytokines that is corelated with the activation of ACC and insula in functional magnetic resonance imaging (fMRI) during a social exclusion task. Many evidences indicate the involvement of peripheral inflammation and neuroinflammation in suicidal behavior (31). Therefore, it can be hypothesized that peripheral inflammation activates the insula *via* interoceptive pathways that in turn modify the brain pain pathways and self-consciousness, inducing psychological pain. In addition, inflammation induced by social stress, infections, or autoimmune diseases could chronically alter the functioning of various neuronal circuits by increasing the production of antineuronal auto-antibodies, promoting neuroinflammation and neurotoxicity through microglia activation, increasing the production of proinflammatory cytokines in the CNS, altering the blood-brain barrier permeability, increasing the activity of self-reactive T cells, and upregulating the transcription of proinflammatory genes (32). This might result in damage to neuronal networks and alteration of self-awareness that could make possible self-aggression.

## Interoceptive Features in Suicide

Studies in the general population and in clinical samples suggest the implication of interoception in suicidal behavior. A systematic review assessed satisfaction with body image, body experience and body ownership (i.e., feelings of detachment from their own body), body sensations and somatic complaints, and interoceptive awareness in adolescents with history of self-injury (33). The main conclusion was that the reported levels of body dissatisfaction, body detachment, somatic complaints, and interoceptive deficits were higher in self-injuring adolescents, especially those who recently attempted suicide. In addition, the longitudinal studies included in this review suggest that disorders in the interpretation of the body sensations are more easily associated with future self-injury compared with body dissatisfaction. Therefore, the association between body and self-injury is not limited to the emotional aspects of the body, but also to the sensation or perception aspects of the body. This is in line with studies suggesting that suicide attempters tend to ignore more their body sensations, and show lower self-regulation using body sensations, and greater interoception deficits (34).

The study of interoception in eating disorders also provides important insights. These patients are at very high risk of suicide (35), and they are chronically engaged in behaviors that harm the physical body, such as self-starvation and self-induced vomiting. Body image distortion and disturbances in interoceptive awareness are among the core symptoms of eating disorders, and interoceptive deficit prospectively predicts greater symptom severity 5–10 years later (36). Anorexia is associated with impaired ability to predict and interpret interoceptive signals, such as feelings of fullness and pain, but also pleasant stimuli, such as an affective touch (37). It has been demonstrated that feeling extraneous from their own body is the experience that discriminates most between people with and without eating disorders. Moreover, it has been proposed that the increased trend to perceive themselves from an external perspective is the way to cope with identity problems (38). Activation of brain regions known to be critical in interoception is increased in patients with history of bulimia nervosa (39). Therefore, interoceptive alterations may make particularly difficult to integrate expectations about homeostatic state changes, ultimately promoting maladaptive behaviors, such as binge eating and food restriction, and possibly self-injury. In people with eating disorders, interoceptive deficits are greater in patients with than without history of suicide attempts (40). In an 8-year longitudinal study, interoceptive deficits at baseline were greater among patients with eating disorders who then attempted suicide during the study (41). Dodd et al. (42) reported in eating disorders a connection between interoceptive deficits and suicide attempts, albeit largely through mediating variables, such as non-suicidal self-injury, and pain tolerance. In patients who attempt suicide, detachment from the body is characterized by interoceptive errors, insensitivity to bodily sensations and a perceived lack of bodily control. Moreover, it is associated with a decreased sensitivity to pain, leading to self-neglect and facilitating self-destructive behavior. Thus, interoceptive deficits might be one of the key factors that increase suicide in these disorders.

Dissociation and depersonalization are experiences that give rise to a feeling of unreality or of being outside their own body and therefore, outside the self. A meta-analysis showed that patients with dissociative disorders were more likely to have attempted suicide or non-suicidal self-injury, compared with patients without dissociative disorder (43). In addition, the scores of dissociation were higher in patients with suicide attempt or with non-suicidal self-injury than in patients without these behaviors. One study examined adolescents with a recent suicide attempt or with a psychiatric disease without previous history of suicide attempt, and healthy controls (44), and found greater psychological pain, lower tolerance for psychological pain, and higher levels of physical dissociation and insensitivity to bodily sensations in suicidal patients. Therefore, in these patients, unbearable mental pain may trigger a process of physical dissociation that manifests itself in insensitivity to physical pain and indifference to the body. The authors concluded that blocking the body awareness and signals makes the body a lifeless object and an easier target for attack.

## Clinical Implications and Avenues for Future Research

Despite these evidences, studies on interoception and suicidal behavior are still in their infancy. Future works should integrate multidimensional assessments (e.g., heartbeat perception tests and specific self-report measures to assess a variety of interoceptive components) to thoroughly elucidate the nature of the associations between suicidal ideation/behavior, pain tolerance, and interoceptive deficits (e.g., attentional biases, distortions of physiological sensitivity, cognitive biases, and insight impairments) (45). Moreover, depression should be taken into account in these analyses because it has been associated with lower interoceptive accuracy (46). Assessing interoception deficits might be part of a multidimensional approach that would help to build suicide risk stratification models, as proposed by Orsolini and colleagues (47).

Focusing on the body and on the evaluation of interoception processes brings interesting perspectives in a field where treatments are lacking. In patients at risk of suicide, the assessment of body awareness and accuracy of interoceptive experiences may be useful to train patients with deficits to be more aware and mindful of their bodily signals (48). People with suicide ideation benefit from mindfulness-based cognitive therapy, an intervention that teaches how to deliberately direct attention to body sensations and to use body sensations to regulate the state of mind (49). It could be hypothesized that neurofeedback strategies (the use of a brain-computer interface to provide feedback about brain functioning) might enable self-regulation of the brain activity in suicide attempters. Indeed, Song et al. (50) recently reported that an event-related potential-based neurofeedback decreases psychological pain in suicide attempters. Similarly, real-time fMRI biofeedback deserves further investigation due to its interest for modulating pain and interoception (51).

Bodily representations are crucial for interacting efficiently and safely with the outside world and for establishing the sense of self as a distinct entity from the rest of the world. Therefore, alterations in the body representations and in the sensations perceived by the body contribute to suicide. Neuroimaging studies show that environmental factors (social stress, social defeat…) and their biological consequences on the body (increased inflammation, neuroinflammation…) can alter the suicide neural networks by increasing sensitivity to psychological pain and negative emotions and also by increasing disconnection from self-awareness. Thus, it is important to concomitantly study body image, body sensations, body awareness and psychological pain, through interoceptive measures to understand the dynamic interactions between body, brain and mind that underlie suicidal behavior.

## Data Availability Statement

The original contributions presented in the study are included in the article/supplementary material; further inquiries can be directed to the corresponding author.

## Author Contributions

All authors contributed to the article and approved the submitted version.

## Conflict of Interest

The authors declare that the research was conducted in the absence of any commercial or financial relationships that could be construed as a potential conflict of interest.
